# Supplementary material for: MetaRibo-Seq measures translation in microbiomes
Source: Nat Commun. 2020 Jun 29;11:3268. doi: 10.1038/s41467-020-17081-z (PMC7324362; doi:10.1038/s41467-020-17081-z)
Supplement: Supplementary file 10 — Supplementary Data 7 [file 41467_2020_17081_MOESM10_ESM.zip › File2/Confidence_High_Taxonomy/260078_out.krona.html]

Javascript must be enabled to view this page.

members
magnitude
magnitudeUnassigned
count
unassigned
taxon
rank

260078\_out

4

superkingdom
2
4

4
1239
phylum

1948061
1
species

SRS011134\_contig\_number\_contig-100\_809.283085

3
186801
class

3
186802
order

186806
3
family

genus
3
1730


SRS049712\_contig\_number\_24223
species
1
2292354


SRS146764\_contig\_number\_26367SRS148253\_contig\_number\_8170
species
2
39490
